# Identification of m5C RNA modification-related gene signature for predicting prognosis and immune microenvironment-related characteristics of heart failure

**DOI:** 10.1186/s41065-025-00454-z

**Published:** 2025-05-22

**Authors:** Zirui Liu, Rui Feng, Ying Xu, Meili Liu, Haocheng Wang, Yu Lu, Weiqi Wang, Jikai Wang, Cao Zou

**Affiliations:** Cardiology Department, First Affiliated Hospital of Soochow University, 188 Shizi Street, Gusu District, Suzhou, Jiangsu Province 215006 China

**Keywords:** m5C, RNA methylation, Heart failure, Bioinformatic analysis

## Abstract

**Background:**

Methylation of RNA is involved in many pathophysiological processes. The roles of N6-methyladenosine (m6A) and N7-methylguanosine (m7G) in heart failure (HF) have been established. However, the impact of 5-methylcytosine (m5C) on HF and its relationship with the immune microenvironment (IME) remains elusive.

**Methods:**

GSE141910 (200 HF, 166 NFDs) was used as training cohort. Focusing on 9 identified m5C differently expressed genes (DEGs), random forests (RF), LASSO logistic regression, and SVM-RFE were employed to identify hub genes. ROC curves were plotted to confirm the predictive value in diagnostic model. ScRNA-seq revealed cell-type-specific m5C regulator expression patterns and HF IME. Hub genes were validated using HF rat models after myocardial infarction (MI) through quantitative reverse-transcription PCR (qRT-PCR) and western blot (WB). Consensus clustering algorithms identified two m5C-related HF subtypes. Single-sample gene-set enrichment analysis (ssGSEA) and CIBERSORT deconvolution algorithm analyzed the IME in HF. Finally, we employed WGCNA and PPI network to find m5C associated key genes and their clinical significance in HF subgroups.

**Results:**

In HF samples, four m5C regulators (NSUN6, DNMT3A, DNMT3B and ALYREF) were greatly upregulated, while five (NOP2, NSUN3, NSUN7, DNMT1 and TRDMT1) were downregulated compared to NFDs in the training set. ALYREF positively correlated with activated NK cells and monocytes, whereas TRDMT1 and NSUN3 showed inverse correlations with these cells. Four hub genes were identified by machine-learning algorithms and all verified by validation model. Single-cell RNA-seq dataset GSE183852 examined the levels of 13 m5C regulators across 11 different cell types in HF. In vivo experiments including qRT-PCR and WB finally identified NSUN6 as the most remarkable regulator. The diagnostic model demonstrated excellent performance in distinguishing between HF and NFDs (AUC 0.869, 95%CI 0.832–0.906). The two m5C subtypes exhibited distinct modification patterns, immune cell infiltration, immune checkpoints, and HLA gene expression. Additionally, 138 differentially expressed genes were uncovered based on m5C subtypes, and GSEA revealed associations with key pathophysiological mechanisms of HF. By using WGCNA and PPI network, three m5C associated key genes (RPS21, RPL36 and RPS19) were identified significantly influencing cardiac function in clinical practice.

**Conclusion:**

HF diagnostic model is developed based on 4 robust m5C RNA modification biomarkers (DNMT3B, NOP2, NSUN6 and DNMT1). Two distinct m5C RNA modification patterns in HF are identified, illustrating different IME characteristics. Our findings underline the significance of m5C regulators in HF, offering new perspectives on HF mechanisms and potential diagnostic and therapeutic strategies.

**Supplementary Information:**

The online version contains supplementary material available at 10.1186/s41065-025-00454-z.

## Background

Heart failure (HF) is a chronic and progressive condition where the heart is unable to effectively pump blood throughout the body, leading to an inadequate supply of blood for physiological demands [1, 2]. This condition is usually caused by various underlying disorders, including hypertension, cardiomyopathy, myocardial infarction (MI), arrhythmias, and valvular diseases [3]. In developing countries, it is estimated that HF affects approximately 1–2% of the general population, indicating that approximately 64.3 million individuals worldwide are currently affected by this condition [4]. Nonetheless, HF pathogenesis remains enigmatic. Immune infiltration seems crucial in HF onset, progression, and prognosis [5]. Exploring alterations in the immune microenvironment (IME) and identifying key regulators in HF may introduce novel ideas for precise diagnosis, prompt intervention, and targeted therapy.

Abundant RNA modifications have been uncovered as key regulators in post-transcriptional regulation, RNA structural stability, and cell metabolism [6]. Among different RNA modifications, RNA methylation is the most common epigenetic modification, mainly including N6-methyladenosine (m6A), N7-methylguanosine (m7G), and 5-methylcytosine (m5C) [7]. Several studies have suggested their vital roles in HF pathogenesis [8]. The dysregulated RNA methylation process is tightly linked to CVDs, especially HF. For example, Ma et al. [9] found that most m7G regulators were differentially expressed between HF patients and normal people and were closely associated with immune responses during HF development. Another bioinformatics analysis study examined the potential role of m6A-related genes in HF [10]. M5C is a common RNA modification found in all types of RNAs, including rRNAs, tRNAs, mRNAs, and non-coding RNAs, across many species [11]. It plays important roles in controlling RNA stability, protein production, and gene activity. Studies have shown that changes in the level of m5C-related regulators are linked to a variety of diseases [12], such as bladder cancer [13] and lung cancer [14]. However, despite the identification of over 10,000 m5C sites [15], the relationship with HF has not been established. Meanwhile, the involvement of m5C in immune responses during HF progression requires more extensive investigation.

**Fig. 1:**
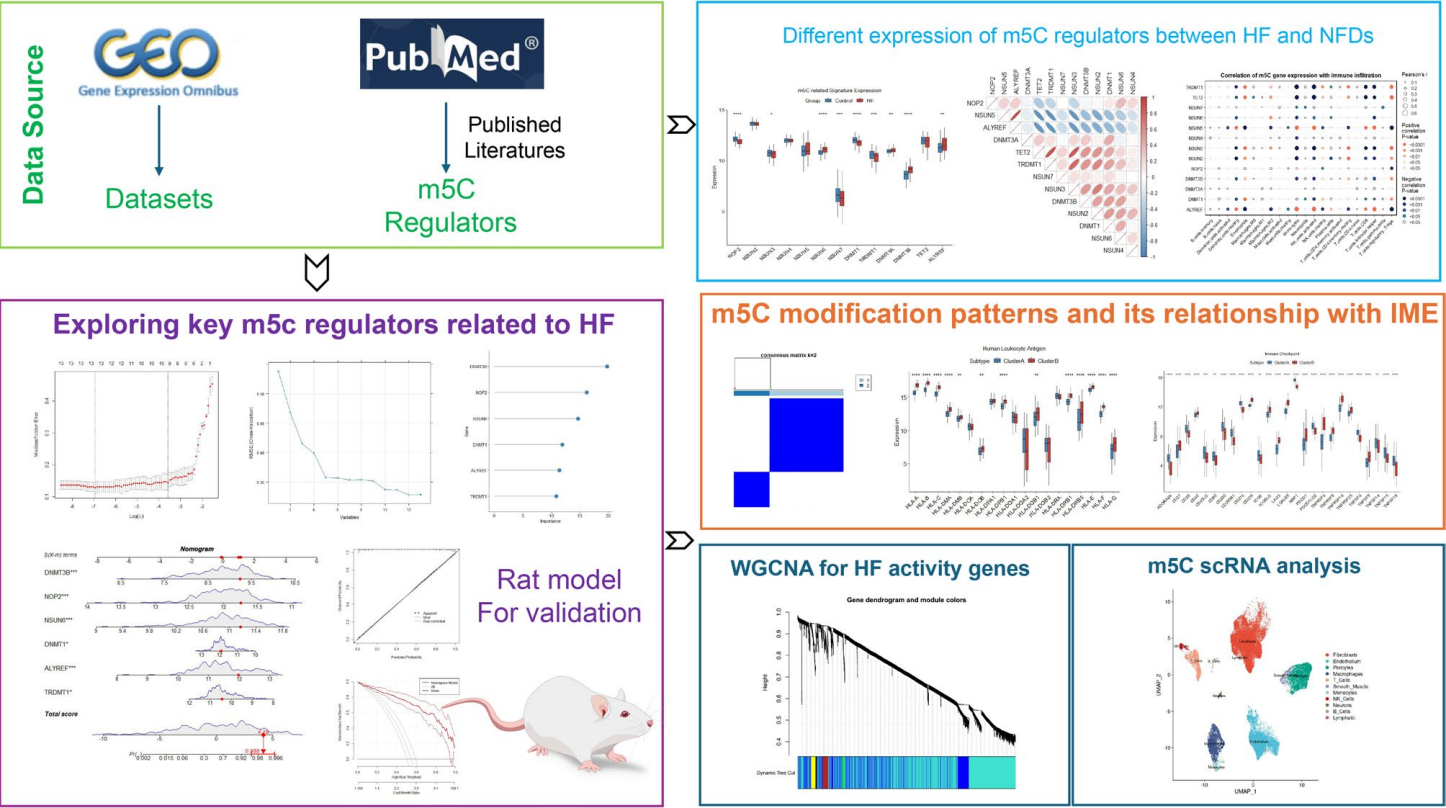
The overview of the study. The study aims to explore the expression of m5C RNA regulators in heart failure (HF) and healthy samples, identify key biomarkers, establish diagnostic models using machine-learning algorithms, and validate the findings through experimental methods. The study also investigates the m5C modification subtypes among HF samples and explores the immune microenvironment (IME) characteristics between these subtypes. WGCNA elucidated hub m5C-related regulators which identified affecting cardiac function. ScRNA sequence also shows the different expression m5C regulators in HF IME

This study initially assessed the modification of 13 m5C regulators in heart failure (HF). Then, SVM, LASSO, and RF were employed to unveil 4 hub m5C regulators and established HF diagnostic model. Furthermore, single-cell RNA sequencing analysis revealed distinct expression patterns of m5C regulators within the IME. Additionally, we established heart failure rat models following myocardial infarction (MI) to validate the expression of hub regulators. Moreover, two molecular subtypes were identified based on these m5C regulators’ expressions. Different immune characteristics and biological functions were observed in these subtypes. Finally, 10 subtype-related hub genes were identified by WGCNA and PPI network. These obtained findings introduce novel biomarkers and perspectives for diagnosis and individualized therapies in HF patients. Figure. 1 shows the overview of this research.

## Methods

### Datasets preprocess

The following criteria were employed for the selection of datasets: (i) the organism was limited to ‘Homo sapiens’; (ii) the study type was classified as ‘Expression profiling by array’; (iii) datasets were required to provide expression data for both heart failure (HF) and non-failing donors (NFDs), with at least three heart samples included; (iv) raw data had to be accessible for reanalysis. Ultimately, seven datasets were incorporated: GSE141910 [16, 17], GSE16499 [18], GSE26887 [19], GSE42955 [20], GSE57338 [21], GSE76701 [22] and GSE79962 [23] were downloaded from GEO database (http://www.ncbi.nlm.nih.gov/geo/). Data preprocessing was conducted using the R package “GEOquery.” Gene probes were annotated according to gene symbols, and probes without corresponding symbols or with multiple symbols were excluded. The maximum value among duplicate symbols was utilized as the representation of gene expression. Using the limma Bioconductor package [24], the datasets underwent preprocessing, including background correction and quantile normalization. We selected GSE141910 as the training set due to its inclusion of the largest number of samples. The remaining six datasets were amalgamated to form a single validation set. To address batch effects arising from different platforms, laboratories, and time points, the Combat algorithm from the sva R package [25] was applied. The results, both before and after batch correction, were visualized through PCA plots (Figure. S1). After correcting for batch effects, the datasets were merged into a single dataset for subsequent validation analyses. To rigorously evaluate our model’s generalizability and mitigate potential overfitting, we performed additional external validation using two independent RNA-seq datasets: GSE46224 [26] and GSE116250 [27]. Detailed information regarding each dataset can be found in Table S1. A total of 13 m5C regulators were identified from prior studies [28], including 11 writers (NSUN2, NSUN3, NSUN4, NSUN5, NSUN6, NSUN7, NOP2, DNMT1, DNMT3A, DNMT3B, TRDMT1,), 1 eraser (TET2), and 1 reader (ALYREF). Single-cell sequencing (scRNA-seq) data containing 5 Transmural LV Apex of HF was downloaded from GSE183852 [29] and analyzed based on the given post-quality control and cells in original papers.

### Alteration analysis of m5C regulators between healthy and HF

The connections among m5C regulators were analyzed using Pearson correlation analysis. The m5C regulators’ levels were compared between NFDs and HF patients via the Wilcoxon test. The STRING database (https://cn.string-db.org/) was employed to assess the interactivity of the m5C regulators via protein-protein interaction (PPI). Using transcriptional data from GSE141910, the CIBERSORT deconvolution algorithm (https://cibersort.stanford.edu/) was used to evaluate the abundance of specific immune cells.

### Screening and validation of m5C diagnostic markers

Key m5C biomarkers for HF were identified using Random Forest (RF), Least Absolute Shrinkage and Selection Operator (LASSO) logistic regression, and Support Vector Machine Recursive Feature Elimination (SVM-RFE). LASSO employs L1 regularization to minimize multicollinearity effects among gene expression features [30]. SVM utilizes kernel-based optimization to maximize margin separation, effectively reducing overfitting risks in high-dimensional omics data [31]. RF robustly handles non-linear relationships and ranks feature importance through ensemble decision trees, making it ideal for prioritizing biologically relevant biomarkers [32]. The RF models utilized the “randomForest” R package, while the LASSO logistic regression employed the “glmnet” R package. Optimization parameters were validated through a ten-fold cross-validation approach, ensuring that the partial likelihood deviation met the predetermined minimum standards. Genes with shared features in three classification models were selected for further analyses. Receiver operating characteristic (ROC) curves were plotted to compute the area under the curve (AUC) to confirm the predictive value. Then calibration curve was drawn to estimate the nomogram’s predictive accuracy. To investigate the biological implications and signaling pathways related to the key regulators of m5C, we performed single-sample gene set enrichment analysis (ssGSEA) using the Gene Ontology (GO) and Kyoto Encyclopedia of Genes and Genomes (KEGG) databases. We categorized the samples into high and low expression groups based on the levels of target genes, which allowed us to identify pathways potentially involved in the pathology of heart failure. For each gene, we ranked the pathways according to the absolute values of their Normalized Enrichment Score (NES), selecting the top five pathways that reached a significance level of *p* < 0.05.

### scRNA-seq data analysis

The scRNA-seq data from GSE183852 was analyzed with Seurat (https://github.com/satijalab/seurat). Cells were filtered for < 200 or > 10,000 genes and mitochondrial gene fragments > 10%. The remaining cells were combined into a gene expression count matrix, and data were normalized and scaled using the NormalizeData() and ScaleData() functions. Following dimension reduction and cluster identification using RunUMAP() and FindClusters() functions, different cell clusters were identified by the SingleR R package. The expression of 13 m5C-related genes was visualized using the featurePlot function.

### Identification of m5C modification patterns

“ConsensusClusterPlus” package identified different m5C clusters through an unsupervised cluster analysis of 9 different expressed genes (DEGs). The “K-Means” algorithm based on “euclidean” distance was utilized, along with 80% resampling and 1000 replications. The optimal k value was determined by the proportion of ambiguous clustering. PCA and heatmap further validated the expression patterns of m5C genes in distinct modification patterns. Besides, the abundance of infiltrating immune cells, immune checkpoints (ICPs), and HLA gene expression were compared in 2 distinct modification patterns via the Wilcox test.

### Biological enrichment analysis for distinct m5C modification patterns

Gene set enrichment analysis (GSEA) determined key pathways and genes between distinct m5C modification patterns. Enrichment analyses checked whether previously defined biological processes were enriched. The enriched pathways were ranked based on their enrichment scores, and those with significance of *P* < 0.05 were chosen for additional analyses. GO analysis identified biological enrichment entries using clusterProfiler R package based on DEGs between distinct m5C modification patterns. The threshold of |logFC|>2 and p value < 0.05 was used for identifying DEGs.

### WGCNA analysis

To identify genes associated with HF disease activity, we utilized Weighted Gene Co-expression Network Analysis (WGCNA) on the gene expression profiles of various HF patient subgroups. We began by selecting the top 25% of genes that showed the highest median absolute deviation across all samples in our integrated dataset, ensuring a diverse and precise dataset for WGCNA. Following this, we included samples that clustered within the established thresholds for further analysis. We computed the adjacency matrix and transformed it into a topological overlap matrix (TOM) using a soft-threshold power β. Using the TOM dissimilarity, we categorized genes into modules with the dynamic tree cut algorithm and merged highly correlated modules (with a correlation greater than 0.8). We then identified co-expressed genes by calculating module membership (MM) and gene significance (GS) within the targeted modules. Additionally, we utilized the STRING database to create PPI networks for the co-expressed genes, applying a threshold weight of 0.9 to define protein interactions. From the PPI network, we extracted the largest connected component and calculated betweenness centrality using Cytoscape. We identified hub genes as the top 10 genes with the highest connectivity degree through the MCC algorithm. Furthermore, we conducted GO and KEGG analyses, visualizing the enrichment bubbles. Lastly, we explored the correlations between the expression profiles of these hub genes and left ventricular ejection fraction (LVEF) in HF patients using the GSE46224 dataset, applying Pearson’s test for this analysis.

### Establishment of HF rat models after MI

Male Sprague–Dawley (SD) rats (220–250 g) were obtained from Shanghai SLAC Laboratory Animal Technology (Animal license number: SCXK (Shanghai) 2022-0004). The HF model induced by MI was developed through surgical ligation of the left anterior descending coronary artery (LAD) [33]. In summary, after inducing deep anesthesia with 2% isoflurane, a left thoracotomy was performed at the third intercostal space to access the heart. The left anterior descending artery (LAD) was then occluded using a 6–0 suture, placed about 2–3 mm below the left atrium. Following successful ligation, the thoracic cavity was closed, and the skin incision was sutured with 4–0 nylon thread. The successful induction of myocardial infarction (MI) was confirmed by observing ST-segment elevation in two or more adjacent chest leads on postoperative electrocardiograms. The rats were placed on an electric blanket to aid in their recovery and were closely monitored during the postoperative period. After the surgery, the rats were kept for 4 weeks under a controlled 12:12 h light-dark cycle at a temperature of 24 °C ± 1 °C and 60 ± 10% humidity, with unrestricted access to standard chow and water. Control (sham) rats underwent the same surgical procedures but without LAD ligation. On the 28th day post-surgery, a total of 12 surviving rats from both the heart failure (HF) and sham groups were euthanized for further analysis. Echocardiographic assessments were performed on the anesthetized rats using a VEVO 2100 imaging system equipped with an M4S transducer. M-mode evaluations of the short-axis view of the heart were utilized to measure the ejection fraction (EF), fractional shortening (FS), and left ventricular internal diameter during diastole and systole (LVIDd and LVIDs).

### Quantitative real-time PCR (qRT-PCR)

Total RNA extraction from cardiac tissues was executed utilizing TRIzol LS (Invitrogen), followed by evaluation of RNA quality and concentration with a NanoDrop ND-1000 analyzer. Reverse transcription was carried out using the GoScript™ Reverse Transcription Mix (Promega). qRT-PCR was performed on an Applied Biosystems QuantStudio 6 apparatus employing SYBR-Green dye (Takara). Data were analyzed using the 2^−(ΔΔCt)^ method with GAPDH as an internal control. Primer sequences are listed in Supplementary Table S3.

### Western blot

Cardiac tissue proteins were extracted using RIPA lysis buffer (Cat. No. WB3100, NCMBiotech). Proteins of varying molecular weights were subsequently separated by Super-PAGE Bis-Tris Gels (4–10%) (Cat. No. LK408, EpiZyme) and transferred onto 0.45 μm PVDF membranes (Cat. No. IPVH00010, EMD Millipore). Following a blocking step with 5% skimmed milk for 1 h at room temperature, membranes were incubated overnight at 4 °C with primary antibodies: GAPDH (1:1000, Cat. No. 10494-1-AP, Proteintech), DNMT3B (1:1000, Cat. No. A11079, ABclonal), and NSUN6 (1:1000, Cat. No. ab307430, Abcam). We designated GAPDH as internal control, consistent with previous studies [34]. The subsequent day, membranes were incubated with secondary antibodies (1:5000, Cat. No. RGAR001, RGAM001, Proteintech) for 1 h at room temperature. Visualization was conducted using ECL (Cat. No. P10060, NCM Biotech), and band intensities were quantified using ImageJ software.

### Statistical analysis

All data analyses were conducted in R programming 4.1.1 (https://www.r-project.org/). Two-group differences were evaluated via Wilcoxon tests (mean ± SD), and *P* < 0.05 denoted statistical differences (ns: no statistical differences, *P < 0.05, **P < 0.01, ***P < 0.001, ****P < 0.0001). The Pearson correlation coefficient was used for correlation analysis. *P* < 0.05 was deemed significant. For qRT-PCR and WB, a t-test (mean ± SEM) was adopted to assess the significance of differences between HF and normal groups.

## Results

### The landscape of RNA m5C regulators between HF and NFDs

**Fig. 2:**
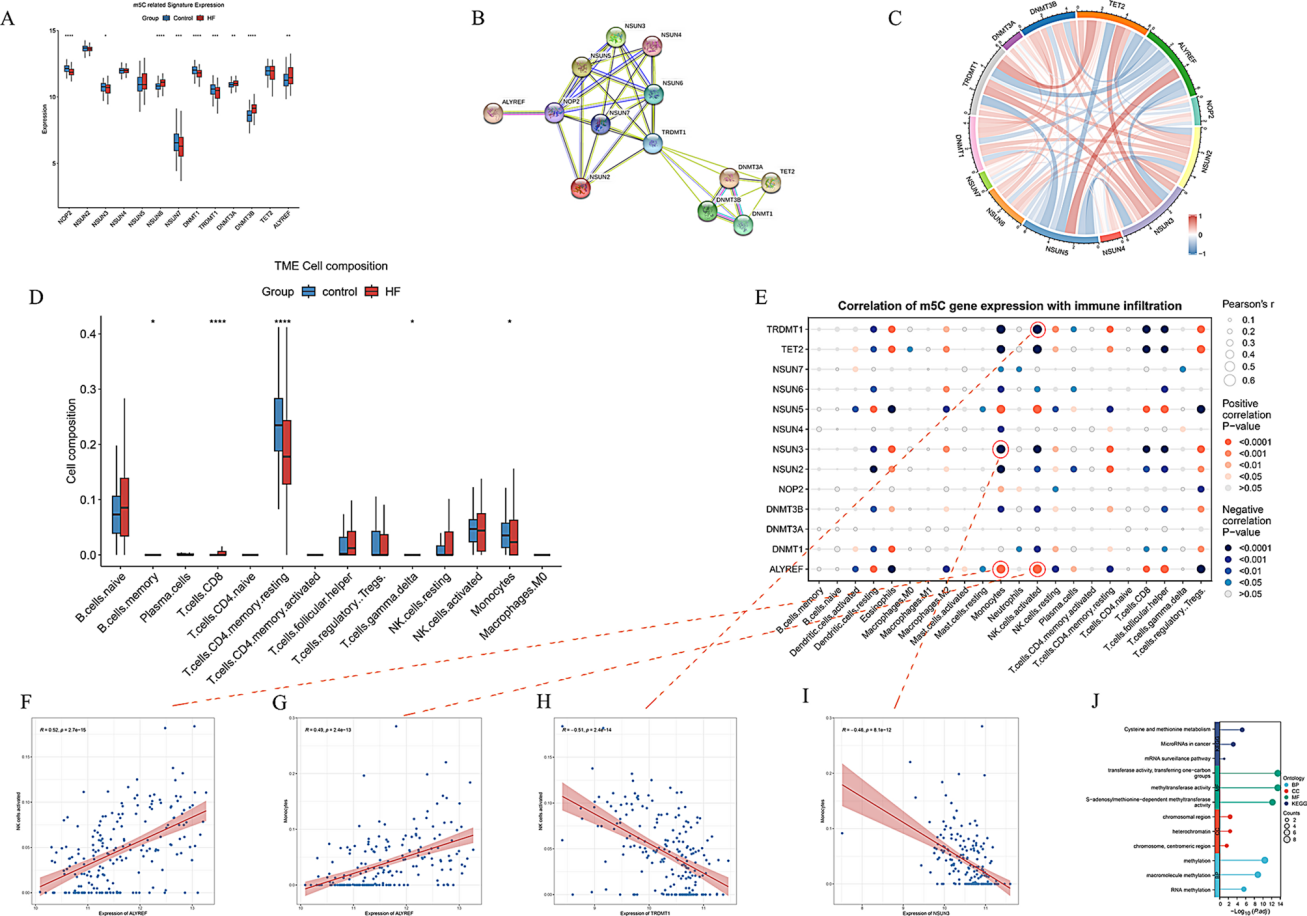
Expression Landscape and Immune Correlations of m5C Regulators in HF and NFDs. (**A**) Differential expression of 13 m5C regulators between HF (*n* = 200) and NFDs (*n* = 166) in GSE141910. (**B**) Protein-protein interaction network revealing strong functional linkages among m5C regulators. (**C**) Correlations among the expression levels of the 13 m5C regulators in HF samples. (**D**) Overview of TME cell composition between HF and healthy samples. (**E**) Immune cell-m5C regulator correlation matrix. (**F**-**I**) Top gene-immune cell correlations: ALYREF vs. NK cells activated (*r* = 0.52, *p* < 0.05, Figure **F**); ALYREF vs. Monocytes (*r* = 0.49, *p* < 0.05 Figure **G**); TRDMT1 vs. NK cells activated (*r*=-0.51, *p* < 0.05, Figure **H**); NSUN3 vs. Monocytes (*r*=-0.46, *p* < 0.05 Figure **I**). Gray shading represents 95% CI. (**J**) GO and KEGG analysis of 9 DEGs

There are 13 m5C RNA methylation-related genes and 9 were found significantly different expressed in the training cohort. NSUN6, DNMT3A, DNMT3B and ALYREF were greatly increased, while NOP2, NSUN3, NSUN7, DNMT1 and TRDMT1 were visibly decreased in HF patients compared with NFDs (Figure. 2 A). The protein-protein interaction (PPI) network was constructed using the STRING database, revealing that these 13 regulatory proteins exhibited robust interconnections, suggesting that these regulators may act collectively as part of a complex functional unit (Figure. 2B). Pearson correlation analysis uncovered a strong positive correlation of ALYREF expression with NSUN5 expression, a strong negative correlation with TET2 and TRDMT1 expression, and strong positive correlations of TET2 with NSUN3 and TRDMT1, and a negative correlation with NSUN5 (Figure. 2 C, Table S2). The abundance of some infiltrating immune cells in HF samples was different from that in NFDs, like B cells memory, T cells CD8, T cells CD4 memory resting, Monocytes, and γδ-T cells (Figure. 2D). According to correlation analysis, m5C-related genes were strongly correlated with infiltrating immune cells (Figure. 2E). ALYREF may be positively correlated with NK cells activated and Monocytes, while TRDMT1 has an opposite trend on NK cells activated, and NSUN3 has an opposite trend on Monocytes (Figure. 2 F-I). Furthermore, GO and KEGG analysis indicated that 9 differently expressed m5C-related genes were enriched in methylation, RNA methylation and methyltransferase activity (Figure. 2 J).

### Identification of key RNA m5C diagnostic markers for HF

**Fig. 3:**
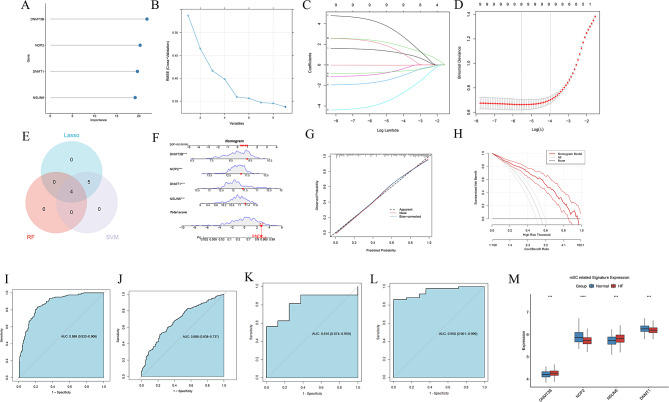
Machine Learning-Based Biomarker Identification and Validation. (**A**) Random forest feature importance ranking. DNMT3B had the highest score. (**B**) SVM-RFE selected 9 features at optimal accuracy. (**C**-**D**) LASSO coefficient profiles (**C**) and 10-fold cross-validation, (**D**) identifying 9 non-zero coefficients. (**E**) Venn diagram showing 4 key biomarkers among the 9 m5C regulators (DNMT1, DNMT3B, NSUN6, NOP2). (**F**) Nomogram of the four-gene m5C regulator diagnostic model for HF probability. (**G**-**H**) Calibration (**G**) and decision curve analysis (**H**) showing clinical utility. (**I**-**J**) ROC curves showing high AUC values in both training (AUC = 0.87) and merged cohorts (AUC = 0.69). (**K**-**L**) ROC curves show high AUC values in independent external cohorts. GSE46224 AUC = 0.82 (**K**), GSE116250 AUC = 0.95 (**L**). (**M**) The boxplot shows the expression of four key regulators in the validation cohort was consistent with the training cohort, including DNMT1, DNMT3B, NSUN6, and NOP2

Three machine learning algorithms identified diagnostic HF biomarkers focusing on 9 different expressed genes (DEGs). The RF model found 4 genes (Figure. 3 A), the SVM-RFE algorithm uncovered 9 genes (Figure. 3B), and the LASSO regression analysis also yielded 9 genes (Figure. 3 C-D). The intersections revealed 4 robust core biomarkers (DNMT3B, NOP2, NSUN6 and DNMT1) (Figure. 3E). Furthermore, a nomogram incorporating the 4 RNA m5C regulators was constructed (Figure. 3 F). The calibration curve manifested minimal deviation between the actual and predicted risk, implying the nomogram’s high accuracy (Figure. 3G). DCA evinced that the nomogram had higher clinical net benefits than all other strategies (Figure. 3 H). Additionally, the diagnostic nomogram increased high AUC values in the training cohort (0.869, 95%CI, 0.832–0.906) and validation cohort (0.688, 95%CI, 0.638–0.737) (Figure. 3I-J), with individual genes also showing strong diagnostic capability (Figure. S2), which validated the solid performance of the diagnostic nomogram. Moreover, this four-gene diagnostic signature demonstrated robust performance in two external validation cohorts GSE46224 (AUC = 0.816, 95%CI 0.674–0.959) and GSE116250 (AUC = 0.950, 95%CI 0.901–0.999), as shown in Fig. 3K-L. In addition, the boxplot of these core biomarkers in validation cohort showed the expression of all 4 hub genes were consistent with training cohort (Fig. 3M).

**Fig. 4:**
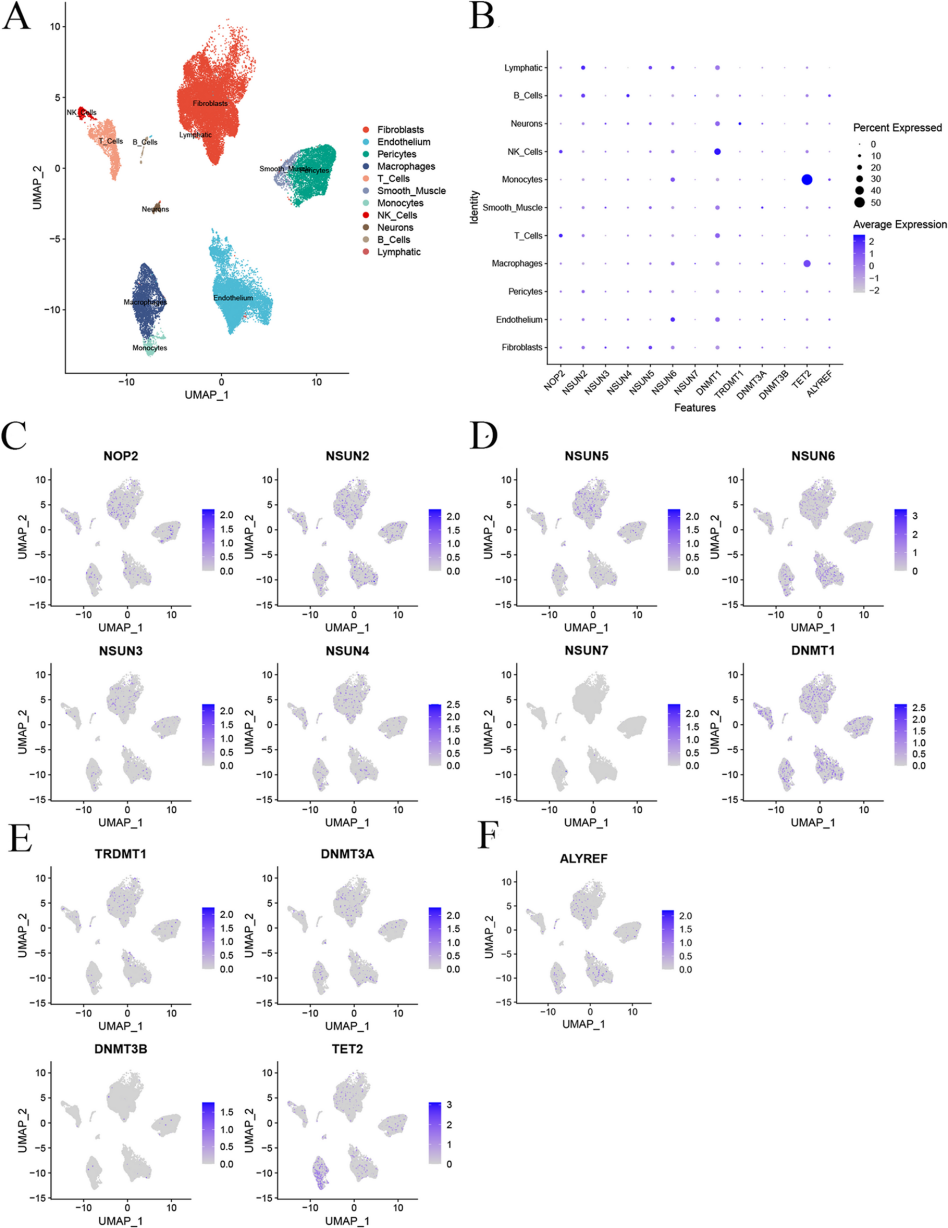
Single-Cell RNA-seq Depicting the Differential Expression of 13 m5C Regulators in HF Immune Microenvironment. (**A**) Major cell types in HF immune microenvironment. (**B**-**H**) Expression differences of NOP2, NSUN2, NSUN3, NSUN4, NSUN5, NSUN6, NSUN7, DNMT1, TRDMT1, DNMT3A, DNMT3B, TET2, and ALYREF in the immune microenvironment

**Fig. 5:**
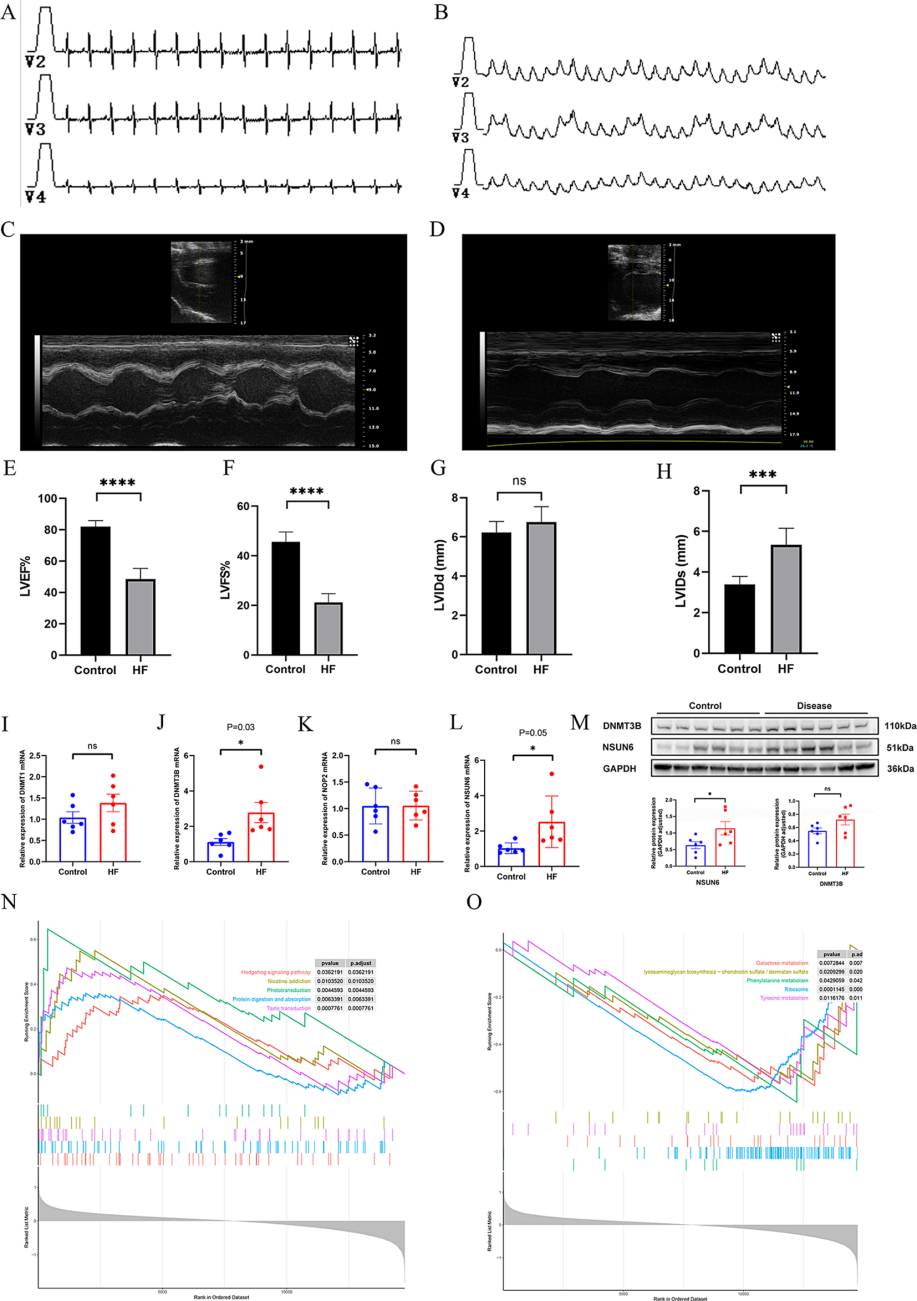
Experimental Validation of the four Hub m5C Regulators. (**A**) ECG before surgery. (**B**) ECG after surgery showing the presence of ST-segment elevation in V2-V4. (**C**-**H**) Significant deterioration in cardiac function indicated by LVEF, LVFS, and LVIDs (except LVIDd). (**I**-**L**) qRT-PCR results for DNMT1, DNMT3B, NOP2 and NSUN6. (**M**) WB results for DNMT3B and NSUN6. (**N**-**O**) ssGSEA analysis for NSUN6

**Fig. 6:**
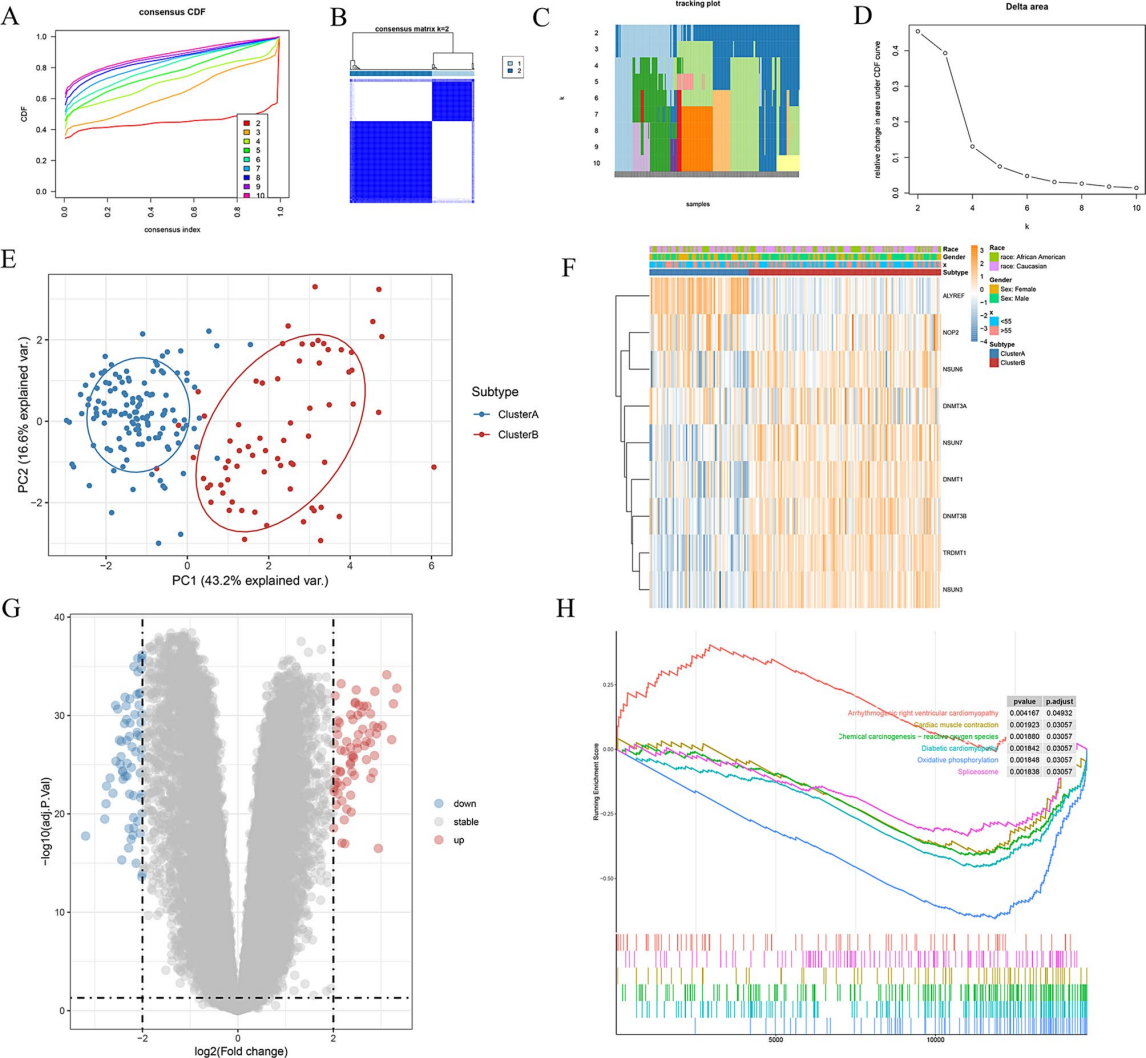
Identification of Two Distinct m5C Modification Subtypes Among HF Samples. (**A**) Consensus clustering model with cumulative distribution function (CDF) for k = 2–10 (k denotes the number of clusters). (**B**) Consensus matrix heatmap defining two subtypes (k = 2) and their correlation area. (**C**) Consensus cluster of items (columns) for k = 2–10 (rows). (**D**) Relative change in the area under the CDF curve for k = 2–10. (**E**) Principal Component Analysis (PCA) showing a significant difference in transcriptomes between the two HF subtypes. (**F**) Expression profiles of m5C DEGs across the two subtypes. (**G**) Volcano plot showing differentially expressed genes (DEGs) in the two subtypes. (**H**) Gene Set Enrichment Analysis (GSEA)

**Fig. 7:**
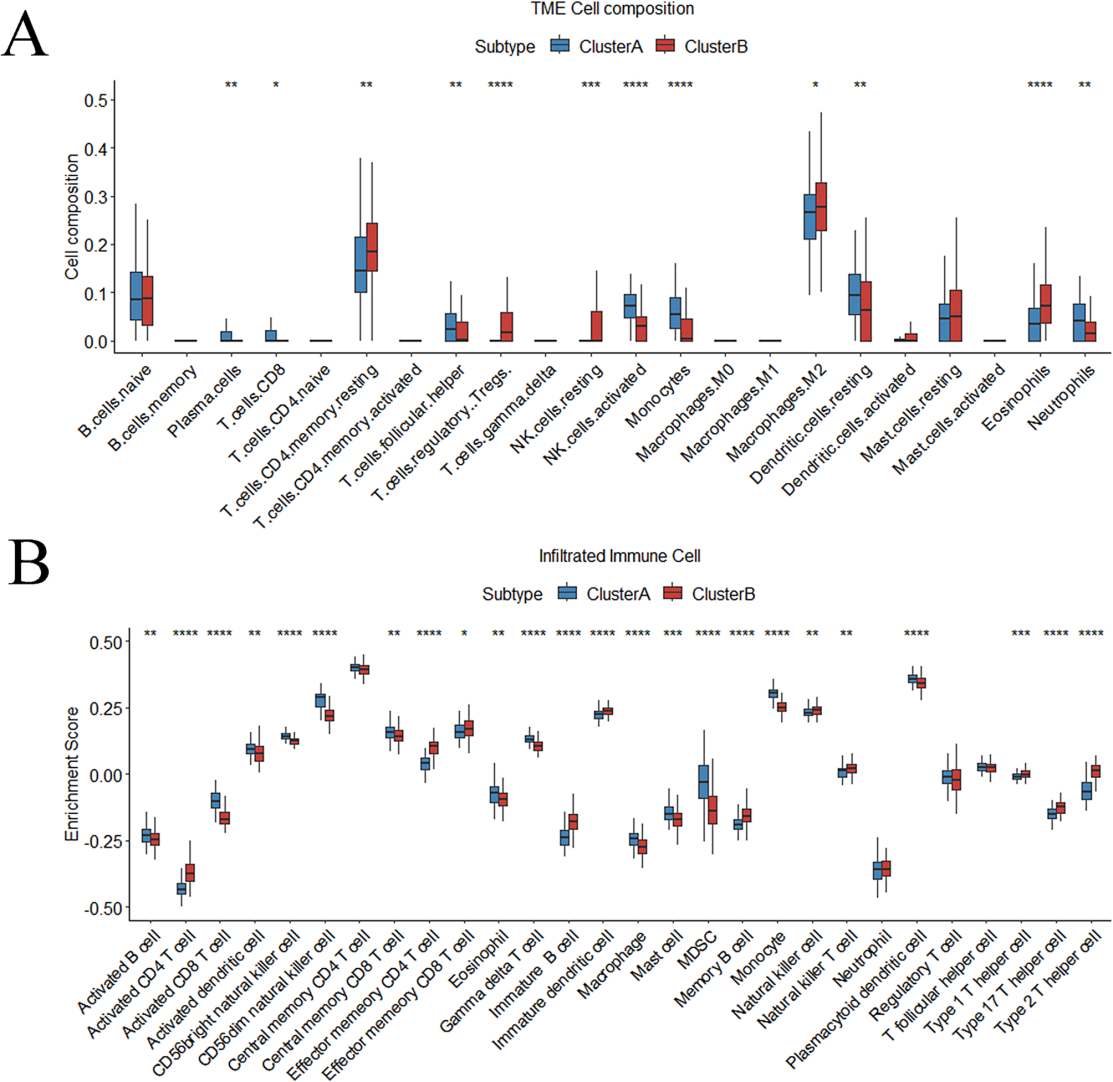
Further Exploration of the infiltrating immune cell Between Two Subtypes. (**A**) Differences in the abundance of each infiltrating immune cell in m5C modification clusters by TME. (**B**) Differences by ssGSEA

**Fig. 8:**
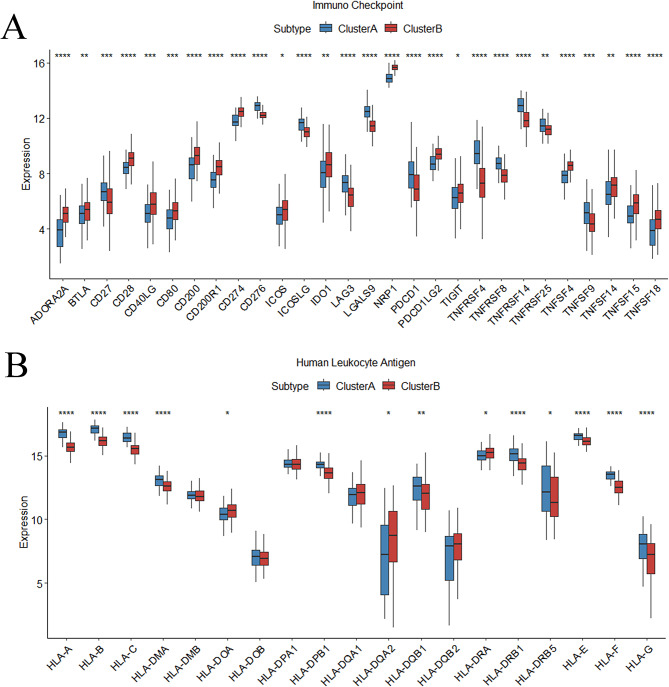
Further Exploration of the Immune Microenvironment Characteristics Between Two Subtypes. (**A**) Immune checkpoint analysis. (**B**) Differences in HLA expression between cluster A and cluster B

### RNA m5c related sc-RNA analysis revealed complicated IME in HF

11 major cell types were identified in HF IME in the GSE183852 dataset (Figure. 4 A), including Fibroblasts, Endothelium, Macrophages, Pericytes, T cells, Smooth Muscle, Monocytes, NK cells, Neurons, B cells, and Lymphocytes, with Fibroblasts being the major type. TET2 expression was high in Monocytes, DNMT1 expression was high in NK cells, and NSUN6 expression was high in Endothelium, while NSUN2 expression was high in B cells and Lymphocytes (Figure. 4B-F).

### Experimental validation of m5C regulators in HF models and pathway analysis

In animal experiments, ECG showed ST-segment elevation in V2-V4, echocardiography showed significant differences between the HF and control groups, which identified the model was successfully established (Figure. 5 A–H). Then, qRT-PCR (Figure. 5I–L) showed DNMT3B and NSUN6 were significant DEGs between HF and control rat samples (*n* = 6), consistent with bioinformatics results, while DNMT1 and NOP2 did not. The results of western blot illustrated the significance of NSUN6 (Figure. 5 M). Altogether, the four-gene m5C-based model exhibited excellent diagnostic capability for HF. Additionally, the ssGSEA analysis of NSUN6 showed Hedgehog signaling pathway was enrichment in HF samples, suggesting its potential role in the progression of HF (Figure. 5 N-O).

### Two RNA m5C modification patterns mediated by 9 DEGs in HF

Consensus clustering analysis determined that k = 2 provided the most stable grouping (Figure. 6 A-D). Thus, 200 HF samples from the GSE141910 were categorized into two distinct groups, Cluster A (*n* = 68) and Cluster B (*n* = 132). PCA plot revealed distinct gene expression patterns between two clusters (Figure. 6E). Distinct m5C-related gene levels were found between two clusters. ALYREF were greatly expressed in Cluster A, while NSUN6, DNMT3A, NSUN7, NSUN3, TRDMT1, DNMT1, and DNMT3B were greatly expressed in Cluster B (Figure. 6 F).

To explore the underlying molecular mechanism between two clusters, we screened out 71 upregulated genes and 67 downregulated genes (Figure. 6G). Furthermore, GSEA analysis showed that Arrhythmogenic right ventricular cardiomyopathy was enriched in Cluster B, while Diabetic cardiomyopathy was enriched in Cluster A (Figure. 6 H).

### Different IME characteristics in two m5C-related clusters

Different immune cells were found between 2 patterns (Figure. 7 A). T cells CD4 memory resting, T cells regulatory Tregs, NK cells resting, Macrophages M2 and Eosinophils were enriched in Cluster B, while Plasma cells, T cells CD8, T cells follicular helper, NK cells activated, Monocytes, Dendritic cells resting and Neutrophils were enriched in Cluster A. The ssGSEA analysis showed significantly different proportions of 24 types of immune cells between subtypes (Wilcoxon test, *P* < 0.05) (Figure. 7B). For example, activated CD4 T cell, effector memory CD4 T cell and Effector memory CD8 T cell were notably higher in Cluster B, while activated B cell, activated CD8 T cell and activated dendritic cell were notably higher in Cluster A. As for ICPs, CD27, CD276, ICOSLG, LAG3, LGALS9, PDCD1, TNFRSF4, TNFRSF8, TNFRSF14, TNFRSF25 and TNFS9 were noticeably elevated, while ADORA2A, BTLA, CD28, CD40LG, CD80, CD200, CD200R1, CD274, ICOS, IDO1, NRP1, PDCD1LG2, TIGIT, TNFSF4, TNFSF14, TNFSF15 and TNFSF18 were noticeably reduced in Cluster A compared with Cluster B (Figure. 8 A). HLA-related genes, including HLA-A, HLA-B, HLA-C, HLA-DMA, HLA-DPB1, HLA-DQB1, HLA-DRB1, HLA-DRB5, HLA-E, HLA-F, and HLA-G were substantially higher in Cluster A (Figure. 8B). These results suggest significant differences in IME between two m5C-related patterns of HF.

### Identification of RNA m5C-related hub genes and their clinical correlation with cardiac function

**Fig. 9:**
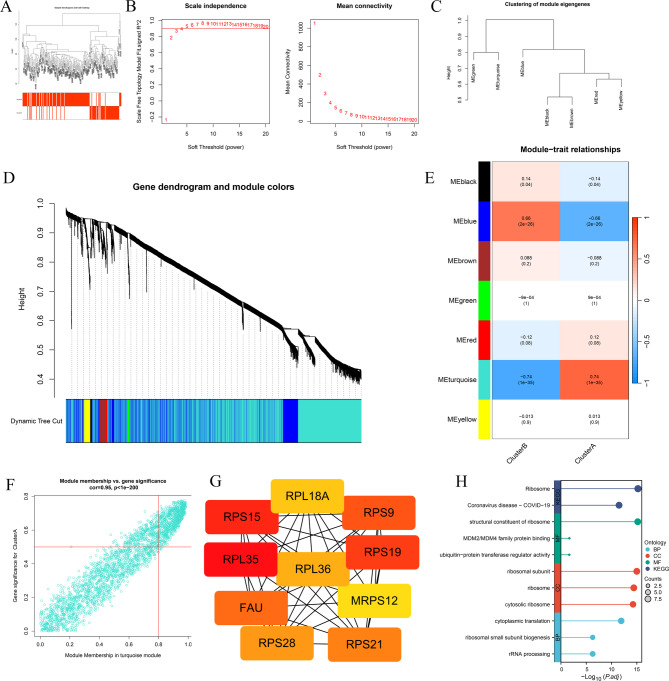
Identification of m5C methylation-associated hub genes in HF. (**A**) Sample clustering was performed using expression data from all HF samples, utilizing the top 25% of variable genes for the WGCNA. (**B**) Analysis of the scale-free topology index and mean connectivity was conducted with soft threshold power ranging from 1 to 20. The red line indicates a scale-free R² value of 0.87, with the soft threshold set at 3. (**C**) Dendrogram of module eigengenes clustering. (**D**) Gene dendrogram created through average linkage hierarchical clustering. Genes were grouped into modules using hierarchical clustering, and modules were merged when their correlation exceeded 0.8. (**E**) Heatmap showing the correlation between module eigengenes and HF gene subgroups. (**F**) Correlation plot illustrating module membership against gene significance for genes in the turquoise module. (**G**) Degree results of genes from the turquoise module. (**H**) GO and KEGG analysis of hub m5C related hub genes

**Fig. 10:**
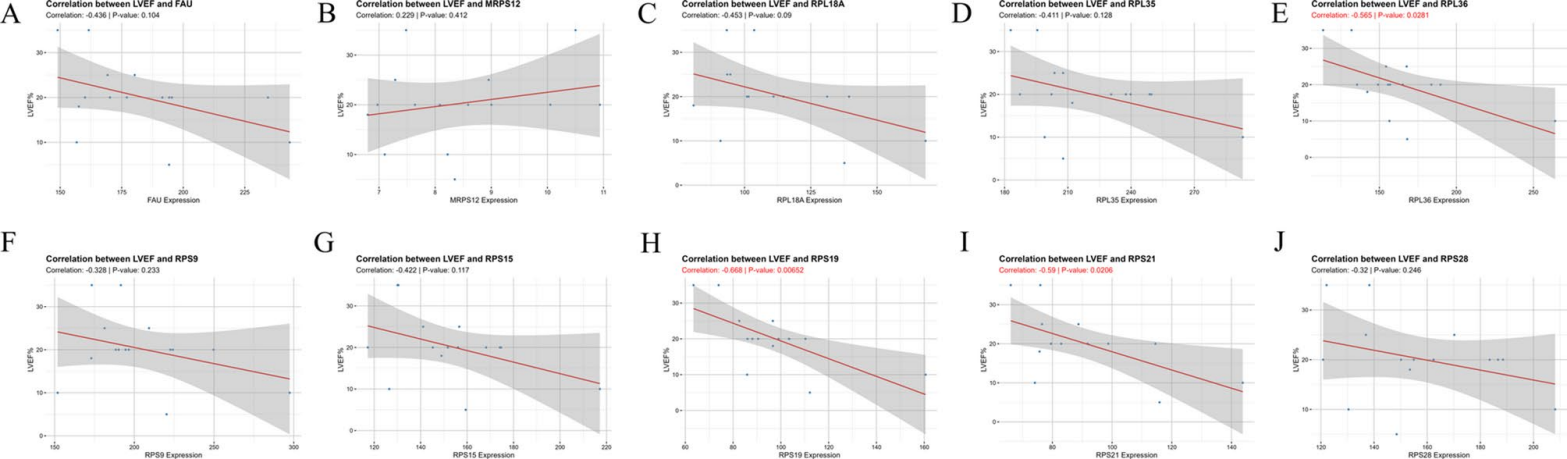
Relationship between m5C markers expression levels and LVEF in HF patients

Figure 9A presents the dendrogram and traits of 200 heart failure (HF) patients based on WGCNA analysis. With a soft threshold of 3, the scale-free topology index (R² = 0.87) was achieved, ensuring higher average connectivity (Figure. 9B). Hierarchical clustering and dynamic branch cutting identified seven distinct modules (Figure. 9 C-D). From the module-trait correlation heatmap, the MEturquoise module exhibited the strongest positive correlation with the m5C gene cluster B in HF (R² = 0.95, *p* < 1e-200), highlighting MEturquoise as a key module (Figure. 9E–F). A total of 859 hub genes from this module were used to construct a protein-protein interaction (PPI) network, and key hub genes were identified using the cytoHubba plugin in Cytoscape. Ultimately, 10 critical m5C markers (RPL18A, RPS9, RPS19, MRPS12, RPS21, RPS28, FAU, RPL35, RPS15, and RPL36) were pinpointed (Figure. 9G). The GO and KEGG analysis showed that these 10 hub genes were significantly enriched in cytoplasmic translation, ribosomal subunit, structural constituent of ribosome, and ribosome (Figure. 9 H). Pearson correlation analysis was performed to assess the relationship between the expression of these markers and left ventricular ejection fraction (LVEF) in HF patients from the GSE46224 dataset. As shown in Fig. 10, RPS21 (*r*=-0.59, *p* = 0.021), RPL36 (*r*=-0.57, *p* = 0.028) and RPS19 (*r*=-0.67, *p* = 0.0065) were negatively associated with LVEF, suggesting that these m5C-related genes may play a role in influencing cardiac function in HF patients.

## Discussion

Emerging evidence has clarified the complex association between HF and immune responses [5, 35–38]. RNA modification, especially RNA methylation, is increasingly recognized in HF [6, 7, 9, 10]. The m5C RNA modification widely exists in eukaryotic RNA molecules [6], including cytoplasmic and mitochondrial rRNAs, tRNAs, mRNAs, and non-coding RNAs. It introduces a methyl group into the fifth carbon atom of cytosine [39] and exerts significant roles in RNA metabolism, tRNA recognition, and stress responses [40]. In recent years, growing studies suggest that m5C contributes to the occurrence and development of diverse diseases. The m5C-related regulators may become potential targets of cancer diagnosis and therapy [41]. Hu et al. [42] found a m5C regulator NSUN2 was upregulated in gastric cancer and promoted the progression of carcinoma. Yu et al. [43]established an excellent diagnostic model for prostate cancer by using m5C genes including NSUN2 and TET3. Zhang et al. [44] established a m5C regulator-related risk model to predict the efficacy of immunotherapy in rectal cancer patients. Recent research also found NSUN2 play a key role in the progression of atherosclerosis through inflammation [45]. NSUN4 deficiency also related to mitochondrial dysfunction and caused cardiomyopathy [46]. However, few articles focus on the global effect of RNA m5C regulators on HF and its relationship with immune microenvironment. Enhanced comprehension of the correlation between m5C RNA modification and HF progression may pave the way for discovering new options for HF diagnosis and treatment. Therefore, we harvested 13 m5C regulators from previous work to systematically explore the modification pattern of m5C regulators in HF based on bulk gene expression datasets.

Firstly, nine significant expression differences were noted in thirteen m5C regulators between HF patients and NFDs, which correlated and interacted with each other, suggesting that these epigenetic regulators may play a key role in HF as a complicated unit. Secondly, we investigated the RNA m5C methylation related immune characteristic by TME between HF patients and NFDs, and observed that adaptive immune systems are activated in heart failure, which is consistent with recent study [47]. The gene-immune cell pairs were also found in HF group, further emphasized the remarkable effect of m5C regulation. Thirdly, different expressions of m5C regulators were identified through scRNA analysis across various cell types within the cardiac microenvironment, providing novel perspectives for HF pathogenesis. Fourthly, a four m5C regulators-based diagnostic signature was constructed using three machine learning algorithms, exhibiting excellent diagnostic value for HF, which further validated in both merged dataset and 2 independent external datasets, and NSUN6 was finally identified as a key regulator in qRT-PCR and WB. Fifthly, two m5C subtypes of HF were revealed through unsupervised cluster analysis, showing distinct m5C modification patterns and immune profiles. Finally, WGCNA was employed to find hub genes in two subtypes and 3 genes were identified to deteriorate cardiac function through ribosome related pathways.

Three machine learning algorithms including RF, LASSO, and SVM were employed to discover hub genes and establish a diagnostic model for HF. The intersections from the above algorithms revealed four vital genes (DNMT3B, NOP2, NSUN6 and DNMT1), which are core diagnostic markers for HF. Importantly, these genes have significant implications in m5C RNA modification-related disease. For example, DNMT1(DNA methyl transferase 1) and DNMT3B keep the RNA methylation stable. Dynamic imbalance of DNMT is linked to atherosclerosis through CD137/NFATc1 signaling [48], and is implicated in many kinds of carcinoma, including breast cancer [49], liver cancer [28], and thyroid cancer [50]. In myocardial ischemia/reperfusion injury, HNEAP knockout restores DNMT1-mediated ATF7 methylation, reduces cardiomyocyte death, and improves cardiac function [51]. Interestingly, in the model of pressure overload-induced cardiac remodeling, Adam et al. [52] demonstrated that DNMT3B inhibition 5-AZA ameliorates cardiac fibrosis, establishing DNMT3B as a potential therapeutic target for HF. Moreover, DNMT3B also has been implicated as a biomarker in CAD progression to AMI [53], highlighting its clinical value in heart failure through m5C-mediated regulation. NOP2 and NSUN6 are both RNA methyltransferases that affect the cell cycle and proliferation activities [54] and show high levels in carcinoma [55, 56] and sepsis [57] and even provide new potential therapeutic approaches for preventing depression [58]. Importantly, through qRT-PCR and WB experiments conducted in SD rat heart tissues, NSUN6 was finally found to be significantly upregulated in HF rats compared with controls. NSUN6 is one of the m5C methyltransferases, which was found to play a critical role in cell proliferation and tumor progression, however its specific effect on HF still needs further exploration. In addition, the 4 gene-based m5C diagnostic model showed an outstanding capability in predicting HF, which was further validated. To sum up with, these findings further strengthen the relevance of these genes in biomarker discovery.

HF, a complex condition, is characterized by immunological dysregulation [59], resulting in inflammation and cellular damage. Recent studies have elucidated the substantial involvement of immune cells, including macrophages [60] and T cells [61], in the pathogenesis and advancement of HF. Recent studies have confirmed the indispensable role of m6A and m7G RNA methylation in HF IME [9, 10]. Targeting immune cells may be promising therapeutic strategies for HF. Then, we hypothesized the similar important role of m5C in immune response-mediated HF process. Consistently, we observed close associations between numerous m5C regulators and immune cells in HF patients. Some studies have found that NK cells and monocytes are destroyed in the IME and induce cardiomyocyte apoptosis in HF animal models [62]. In this study, ALYREF-NK cells and ALYREF-Monocytes pairs strongly showed positive correlation among m5C regulator-immune cell pairs. It is worth noting that ALYREF expression also showed significant positive correlation with monocyte/macrophage infiltration in bladder cancer, suggesting a potential role of m5C regulators in shaping tumor immune microenvironments [13]. Additionally, DNMT1-mediated epigenetic regulation of immune responses may critically impact cardiovascular disease pathogenesis, given its close association with immune signaling pathways, such as T-cell receptor (TCR), and B-cell receptor (BCR) [63]. Collectively, these findings indicate that m5C regulators may contribute to HF progression through immune-related pathways.

In this study, we identified 11 major cell types within heart failure (HF) immune microenvironments, with fibroblasts as the dominant cell type. Our findings show that TET2, DNMT1, and NSUN family methylation regulators exhibit distinct cell-specific expression patterns, with TET2 highly expressed in monocytes, DNMT1 in NK cells, NSUN6 in endothelial cells, and NSUN2 in both B cells and lymphocytes. These differential expression patterns suggest that RNA methylation regulators may contribute to cell-type-specific roles in HF pathogenesis, potentially influencing cellular functions and interactions within the immune microenvironment.

Two m5C patterns were revealed to explore the biological features of 13 m5C regulators in HF. Importantly, unique immune characteristics (immune cells, ICPs, and HLA gene expression) were found between the two clusters. In brief, 24 kinds of immune cells, 28 ICPs, and 14 HLA genes showed differences between the two subtypes. Interestingly, different types of T cells in 2 subtypes imply their indispensable roles in HF [59]. For example, the proportion of CD8 positive T cells, plasma, NK cells activated and Monocytes are significantly higher in Cluster A than Cluster B. Conversely, Macrophages M2, which identified reducing inflammation, is higher than Cluster A. Studies has elucidated that sustained immune and inflammatory responses always resulted in cardiac remodeling and brought adverse clinical outcomes [47]. So, Cluster A may be a group which has worse prognosis. In recent years, immune checkpoints (IC) have received more attention as a potential target for end stage of HF. Mice with knocked out of B7 (CD276) had lower cardiac inflammation after TAC, consistent with using B7 blocker called CTLA4 immunoglobulin [64, 65]. It may be beneficial for Cluster A if using CTLA4 immunoglobulin for a better cardiac function. As a result, these differences highlight the complex biological function of the immune system in HF progression, which may help us better understand the mechanism and explore m5C-mediated HF immune therapy strategies. Additionally, we further investigated 138 m5C DEGs. GO and KEGG-GSEA analysis showed m5C methylation widely presented in Arrhythmogenic right ventricular cardiomyopathy, Cardiac muscle contraction, Oxidative phosphorylation, Diabetic cardiomyopathy, and Spliceosome, indicating the key role of m5C in HF. And we further used WGCNA and Cytohubba to find ten hub genes related to m5C subtypes. GO and KEGG analysis revealed these 10 genes are enriched in ribosome, RPL36, RPS19 and RPS21 were linked with a worse LVEF when expressed higher. However, little is known about these ribosome related genes and its relationship with HF. On the other hand, studying ribosomes may provide a new direction for the treatment of HF.

Our study is the inaugural investigation into the potential involvement of m5C RNA methylation modifications in HF. However, several limitations exist. Firstly, the reliance on publicly available datasets may introduce inherent biases, highlighting the need for future prospective studies to validate our findings. Secondly, despite support from animal experiments, further external validation from clinical cohorts is essential to confirm their relevance. Thirdly, additional in vitro/in vivo experiments are needed to demonstrate the direct function about how m5C regulators influence HF progression. Additionally, comprehensive experimental research is crucial to delineate the expression profiles and molecular mechanisms of the identified m5C genes. Lastly, there is a dearth of downstream evidence regarding m5C modifications between HF patients and healthy individuals. The mechanisms and pathways through which m5C methylation influences myocardial energy metabolism and immune infiltration remain to be explored. Therefore, further investigation is warranted to address these gaps.

## Conclusion

In conclusion, our study has shed light on the complex regulatory mechanisms of RNA m5C methylation modifications and the IME in HF, potentially providing new perspectives for HF diagnosis and therapeutics.

## Electronic supplementary material

Below is the link to the electronic supplementary material.

Supplementary Material 1

Supplementary Material 2

Supplementary Material 3

## Data Availability

Data is provided within the manuscript.

## Abbreviations

m5C: 5-methylcytosine
m6A: N6-methyladenosine
m7G: N7-methylguanosine
HF: Heart failure
NFDs: Non-failing donors
CVD: Cardiovascular diseases
ROC: Receiver operating characteristic
AUC: Area under the curve
qRT-PCR: Reverse-transcription polymerase chain reaction
WB: Western blot
GO: Gene Ontology
HLA: Human leukocyte antigen
ICPs: Immune checkpoints
IME: Immune microenvironment
KEGG: Kyoto Encyclopedia of Genes and Genomes
NES: Normalized enrichment score
PAC: Proportion of ambiguous clustering
WGCNA: Weighted gene co-expression network analysis
TOM: Topological overlap matrix
MM: Module membership
PPI: Protein-protein interaction
ssGSEA: Single-sample gene set enrichment analysis
NSUN2: NOP2/Sun RNA methyltransferase 2
NSUN3: NOP2/Sun RNA methyltransferase 3
NSUN4: NOP2/Sun RNA methyltransferase 4
NSUN5: NOP2/Sun RNA methyltransferase 5
NSUN6: NOP2/Sun RNA methyltransferase 6
NSUN7: NOP2/Sun RNA methyltransferase 7
NOP2: NOP2 nucleolar protein
DNMT1: DNA methyltransferase 1
DNMT3A: DNA methyltransferase 3 A
DNMT3B: DNA methyltransferase 3B
TRDMT1: tRNA aspartic acid methyltransferase 1
TET2: Tet methylcytosine dioxygenase 2
ALYREF: Aly/REF export factor
RPL36: Ribosomal Protein L36
RPS21: Ribosomal Protein S21
RPS19: Ribosomal Protein S19
