# Supplementary material for: Identification of m5C RNA modification-related gene signature for predicting prognosis and immune microenvironment-related characteristics of heart failure
Source: Hereditas. 2025 May 22;162:83. doi: 10.1186/s41065-025-00454-z (PMC12096717; doi:10.1186/s41065-025-00454-z)
Supplement: Supplementary file 1 — Supplementary Material 1 [file 41065_2025_454_MOESM1_ESM.docx]

**Table S1** Details of GEO datasets.

| **GSE number** | **Platform** | **HF samples** | **Normal samples** | **Group** | **Data type** |
| --- | --- | --- | --- | --- | --- |
| GSE141910 | GPL16791 | 200 | 166 | Training cohort | Microarray |
| GSE16499 | GPL5175 | 15 | 15 | Validation cohort |  |
| GSE26887 | GPL6244 | 19 | 5 |  |  |
| GSE42955 | GPL6244 | 24 | 5 |  |  |
| GSE57338 | GPL11532 | 177 | 136 |  |  |
| GSE76701 | GPL570 | 4 | 4 |  |  |
| GSE79962 | GPL6244 | 11 | 40 |  |  |

**Table S2** m5C gene correlations.

|  | ALYREF | DNMT3A | DNMT3B | DNMT1 | NOP2 | NSUN2 | NSUN3 | NSUN4 | NSUN5 | NSUN6 | NSUN7 | TET2 | TRDMT1 |
| --- | --- | --- | --- | --- | --- | --- | --- | --- | --- | --- | --- | --- | --- |
| ALYREF | 1 | -0.14395 | -0.59211 | -0.54404 | 0.24771 | -0.64382 | -0.7812 | -0.20701 | 0.939181 | -0.45491 | -0.27077 | -0.80883 | -0.83806 |
| DNMT3A | -0.14395 | 1 | 0.268421 | 0.414908 | -0.06031 | 0.067174 | 0.206129 | 0.020424 | -0.13183 | -0.08049 | -0.01298 | 0.261784 | 0.14105 |
| DNMT3B | -0.59211 | 0.268421 | 1 | 0.500839 | -0.04005 | 0.476045 | 0.511812 | 0.057386 | -0.51096 | 0.47816 | 0.143147 | 0.533286 | 0.535442 |
| DNMT1 | -0.54404 | 0.414908 | 0.500839 | 1 | 0.101935 | 0.515572 | 0.452181 | -0.0338 | -0.50653 | 0.415218 | 0.160514 | 0.435775 | 0.474437 |
| NOP2 | 0.24771 | -0.06031 | -0.04005 | 0.101935 | 1 | -0.03828 | -0.32831 | 0.196074 | 0.224527 | 0.34653 | 0.00437 | -0.41285 | -0.32459 |
| NSUN2 | -0.64382 | 0.067174 | 0.476045 | 0.515572 | -0.03828 | 1 | 0.664609 | 0.360626 | -0.6702 | 0.479504 | 0.06747 | 0.543959 | 0.615053 |
| NSUN3 | -0.7812 | 0.206129 | 0.511812 | 0.452181 | -0.32831 | 0.664609 | 1 | 0.284967 | -0.82487 | 0.334369 | 0.153908 | 0.850892 | 0.786371 |
| NSUN4 | -0.20701 | 0.020424 | 0.057386 | -0.0338 | 0.196074 | 0.360626 | 0.284967 | 1 | -0.23981 | 0.189556 | 0.004822 | 0.076297 | 0.104358 |
| NSUN5 | 0.939181 | -0.13183 | -0.51096 | -0.50653 | 0.224527 | -0.6702 | -0.82487 | -0.23981 | 1 | -0.40955 | -0.24939 | -0.84137 | -0.84479 |
| NSUN6 | -0.45491 | -0.08049 | 0.47816 | 0.415218 | 0.34653 | 0.479504 | 0.334369 | 0.189556 | -0.40955 | 1 | 0.037443 | 0.198311 | 0.317713 |
| NSUN7 | -0.27077 | -0.01298 | 0.143147 | 0.160514 | 0.00437 | 0.06747 | 0.153908 | 0.004822 | -0.24939 | 0.037443 | 1 | 0.211165 | 0.185405 |
| TET2 | -0.80883 | 0.261784 | 0.533286 | 0.435775 | -0.41285 | 0.543959 | 0.850892 | 0.076297 | -0.84137 | 0.198311 | 0.211165 | 1 | 0.882571 |
| TRDMT1 | -0.83806 | 0.14105 | 0.535442 | 0.474437 | -0.32459 | 0.615053 | 0.786371 | 0.104358 | -0.84479 | 0.317713 | 0.185405 | 0.882571 | 1 |

**Table S3** Primer sequences for qRT-PCR.

| **Gene Symbol** | F | R |
| --- | --- | --- |
| GAPDH | GACATGATGCCTGGAGAAAC | AGCGGCAGGATGCCCTTTAGT |
| NOP2 | GTTAAAGCTGTCTGAGTTGA | GTGGCATTCCGAAGGGTGAT |
| DNMT1 | CCTGGGGCCAACCAATCAGT | GCTGGGCCCTCCATCAGAAGT |
| DNMT3B | AGGAAGACAAGAACTTGGCG | ATCGCTGGGTACAACTTGGG |
| NSUN6 | ATCCCTCCAGTTCCGATGCT | ATGCTGATAGGAGGCTTTAC |
