# Supplementary material for: Identification of m5C RNA modification-related gene signature for predicting prognosis and immune microenvironment-related characteristics of heart failure
Source: Hereditas. 2025 May 22;162:83. doi: 10.1186/s41065-025-00454-z (PMC12096717; doi:10.1186/s41065-025-00454-z)
Supplement: Supplementary file 2 — Supplementary Material 2 [file 41065_2025_454_MOESM2_ESM.docx]

The membrane was cut into strips to probe for the target protein.


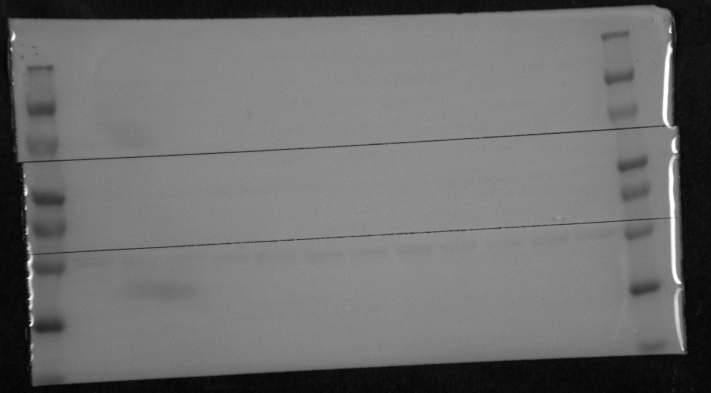


1. Whole membrane


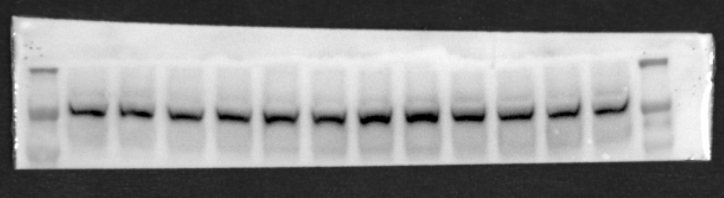


B. DNMT3B protein


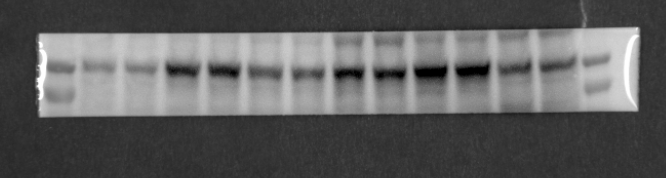


C. NSUN6 protein


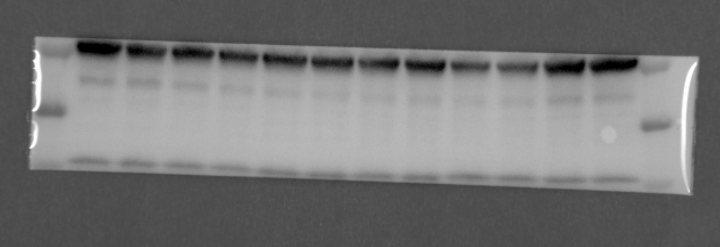


D. GADPH protein
