# Supplementary material for: Identification of m5C RNA modification-related gene signature for predicting prognosis and immune microenvironment-related characteristics of heart failure
Source: Hereditas. 2025 May 22;162:83. doi: 10.1186/s41065-025-00454-z (PMC12096717; doi:10.1186/s41065-025-00454-z)
Supplement: Supplementary file 3 — Supplementary Material 3 [file 41065_2025_454_MOESM3_ESM.docx]

**Figure S1** Data normalization. (A) PCA plot before data integration. (B) PCA plot after data integration.


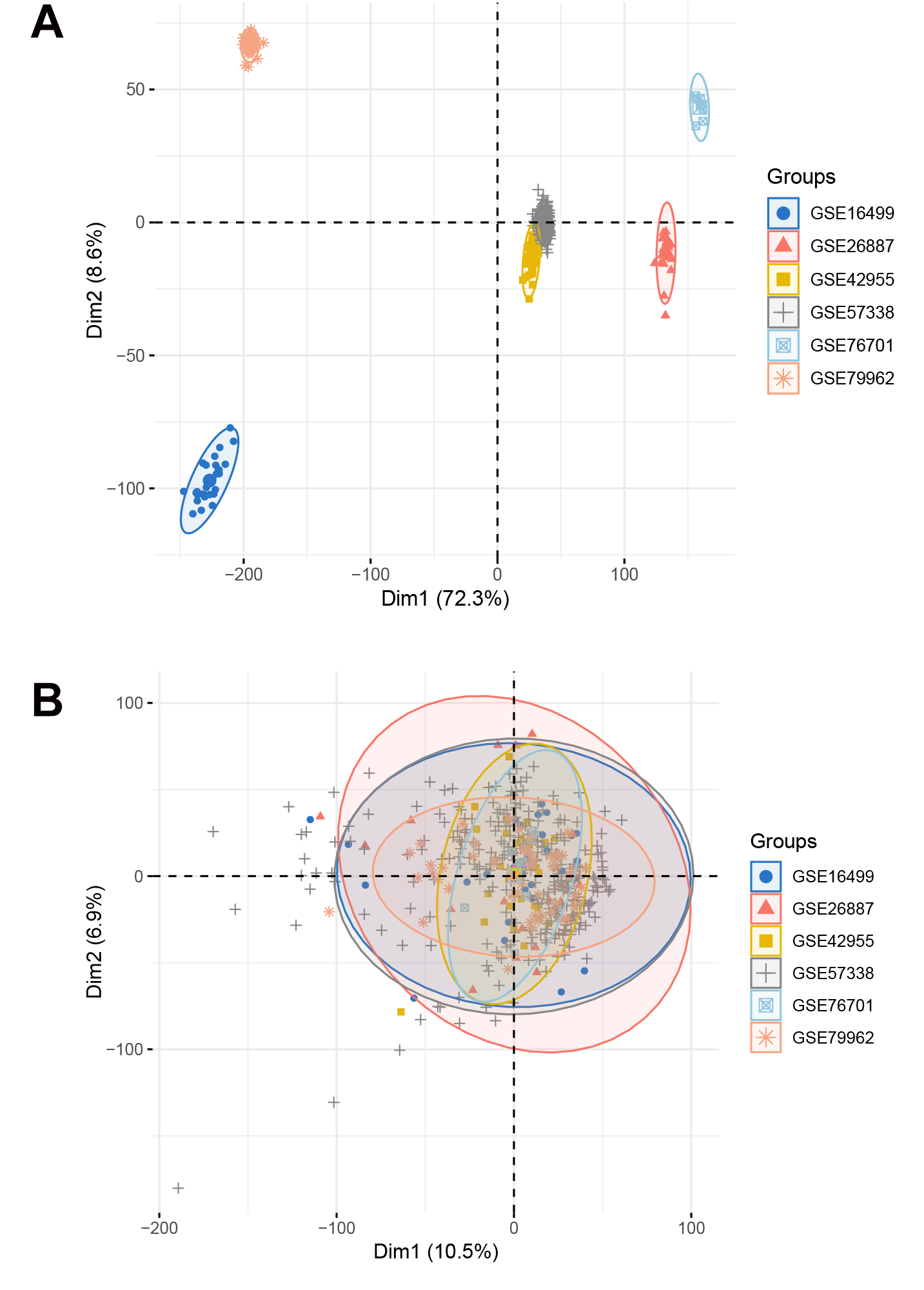


**Figure S2** ROC curves show high AUC values in hub genes. (A) Training cohort. (B) Validation cohort (6 datasets merged).


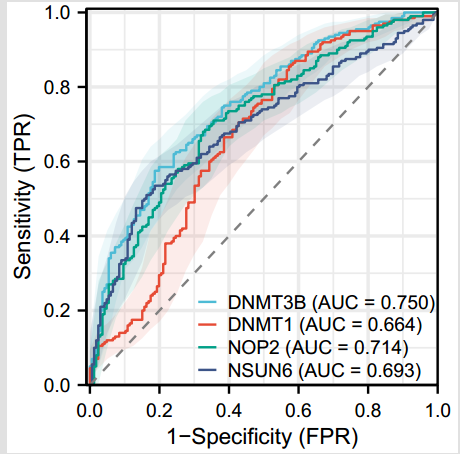

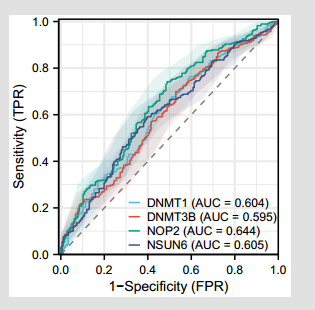


**A**

**B**
